# Health System Leadership for Psychological Health and Organizational Resilience During the COVID-19 Pandemic: Protocol for a Multimethod Study

**DOI:** 10.2196/66402

**Published:** 2025-05-28

**Authors:** Sonia Udod, Ibrahim Jahun, Pamela Elizabeth Baxter, Jaason M Geerts, Maura MacPhee, Gayle A Halas, Greta G Cummings, Suzanne Marie Gagnon

**Affiliations:** 1 Helen Glass Centre for Nursing College of Nursing University of Manitoba Winnipeg, MB Canada; 2 School of Nursing McMaster University Hamilton, ON Canada; 3 The Canadian College of Health Leaders Ottawa, ON Canada; 4 Cambridge Judge Business School University of Cambridge Cambridge United Kingdom; 5 Telfer School of Management University of Ottawa Ottawa Canada; 6 School of Nursing University of British Columbia Vancouver, BC Canada; 7 Rady Faculty of Health Sciences University of Manitoba Winnipeg, MB Canada; 8 Faculty of Nursing University of Alberta Edmonton, AB Canada; 9 Asper School of Business University of Manitoba Winnipeg, MB Canada

**Keywords:** health system leadership, COVID-19, psychological health, organizational resilience, crisis leadership, crisis model

## Abstract

**Background:**

Since the World Health Organization declared COVID-19 a global pandemic, health systems and health system leaders have faced unprecedented challenges through the various stages of the crisis. Canada and other health systems were largely ill-prepared to handle this crisis. The longevity of the pandemic has profoundly affected health care systems and compounded the rates of negative psychological outcomes in health systems’ leaders and staff, rates of emotional exhaustion, and burnout.

**Objective:**

The purpose of this study is to investigate the experiences of health system leaders and nurses during COVID-19 and to develop recommendations to inform pre-, during-, and postcrisis leadership strategies and practices for health system leaders, which address leaders’ and nurses’ psychological health and well-being, as well as organizational resilience.

**Methods:**

A 3-year multimethod approach will be adopted and include a qualitative exploratory inquiry informed by Geerts’ 4-stage framework of imperatives for health system leaders to guide data collection and analysis. We will then conduct semistructured individual interviews with health system leaders in 3 provinces in Canada and hold focus group interviews (FGIs) with nurses from the same organizations. Data from the interviews and FGIs will be integrated to determine how health system leaders promoted their own health and how their leadership shaped nurses’ psychological health and contributed to building organizational resilience. We will engage knowledge users using a nominal group technique in a 1-day forum to discuss how findings can be applied in professional contexts. We will conduct a thematic analysis of the aggregated data to identify and analyze themes to provide an interpretive explanation of health system leaders’ experiences and organizational resilience during the COVID-19 pandemic, and how the leaders promoted nurses’ psychological health and well-being. The protocol has been reviewed and approved by the University of Manitoba institutional review board (IRB), the University of Alberta IRB, and McMaster University Ontario IRB.

**Results:**

As of September 6, 2024, this study has made significant progress. Data collection has been completed for individual interviews with health leaders in Alberta and Manitoba, and has commenced in Ontario. FGIs will be completed by the fall of 2025, data integration in early 2026, nominal group technique in the spring of 2026, and the final report will be written in the summer of 2026.

**Conclusions:**

The findings will support practices that health system leaders can implement to foster their own and nurses’ psychological health and well-being and build organizational resilience. The benefits of this study aim to include evidence for effective health system leadership and support for nurses, crisis preparedness, and lessons from the pandemic to address leadership practices to operationalize the imperatives within the 4 stages of the crisis model.

**International Registered Report Identifier (IRRID):**

DERR1-10.2196/66402

## Introduction

### Background

Since the World Health Organization declared COVID-19 a global pandemic [1], health systems and health system leaders have faced unprecedented challenges through the various stages of the crisis. Dr Theresa Tam, Canada’s Chief Public Health Officer, warned of the threat of pandemics and the need to prepare more than 15 years before COVID-19 [2], and yet, health systems worldwide, including Canada’s, were largely ill-prepared to respond effectively to this crisis [3-5]. COVID-19 placed novel and extreme demands on health care systems and their leaders by exceeding regional and organizational capacity for detection and outbreak containment, rationing of the supply chain and required resources, infodemic management, and the health care workforce to maintain operations [6-8].

Given the certainty of future pandemics and health care crises, there is a pressing need for immediate action to bolster national, regional, and organizational emergency preparedness and to safeguard the welfare of the health care workforce [9]. Nurses make up a significant proportion of the health care workforce worldwide, and in Canada and elsewhere, this profession was in crisis before the COVID-19 onset [8]. A global shortage of almost 6 million nurses was driven by an ageing population and increased care needs, retirements, high turnover, and low retention, including many leaving the profession altogether, and an ageing workforce [9]. One Canadian study found 93% of nurses had symptoms of burnout and almost 1 in 3 had clinical levels of burnout requiring medical attention [10]. The pandemic has exacerbated this situation for nurses, causing further deterioration of their mental health [11] and rates of burnout [12-16]. This is attributable to the demoralizing work conditions in which nurses have worked with severely inadequate resources and overwhelming workloads at the front lines of care [8,9]. A survey of 1705 frontline nurses in Canada in 2020 found high chronic fatigue, lower work satisfaction, and higher intention to leave their organization for those caring for patients with COVID-19, which resulted in poor quality of care provision [17-19]. Similarly, for health system leaders, despite their extensive experience navigating forces influencing organizational change, the prolonged and chaotic COVID-19 situation exposed leaders to unique leadership challenges [20]. The longevity of the pandemic profoundly affected health care.

Leadership is critical in a crisis and affects organizational performance and resilience [14,21-23]. Organizational resilience is a multidimensional concept that refers to the ability of individuals and groups to maintain operations and stability during situations of uncertainty, including how an organization reacts to and recovers from major disruptions, with minimal disturbance [24-27]. The leader’s role is to anticipate potential problems in the environment, secure resources, make and guide others in making good decisions regarding priorities and strategies, and take action, and minimize the negative impact of a disturbance [28]. These are key leadership capabilities that increase the organization’s awareness and adaptive capacity [29]. In a crisis, despite intense pressure, complexity, and uncertainty, leaders must be decisive, obtain and allocate stretched resources, and communicate clearly with employees, often under a spotlight [30-32].

Few empirical studies exist on health system leadership in crises, and what is available is often either limited to anecdotal summaries of lessons learned by senior leaders and their corresponding suggested strategies [30,33,34] or is restricted to disaster preparedness [26] and acute emergency response [27]. Although COVID-19 involved waves of intense emergency periods, which required a decisive reaction, it also presented distinctly different crisis subcontexts, such as the escalating warning period before the first local impact, as well as postemergency but prefinal resolution phases of eerily uncertain length [28]. The unique characteristics and demands facing leaders in each of these situations require customized leadership priorities and strategies. To the best of our knowledge, there is a dearth of evidence-informed leadership guidance for each stage of a crisis.

The scale of the COVID-19 pandemic and the corresponding challenges it presented, such as the infodemic, supply chain inefficiencies, and massive leader and staff shortages, absenteeism, burnout, and turnover, severely overwhelmed hospitals, many of which were notably overburdened before the pandemic [35,36]. The pandemic has taken a toll on health system leaders and nurses, including on the deterioration of nurses’ well-being and their capacity to be engaged and provide compassionate care, which compromises the quality of care that they can provide to patients, and their ability to tackle the backlog of surgeries and other services that have been paused over the past 5 years. Consequently, the need to improve leader and nurse psychological well-being [9,13,35,37], and the need for organizational resilience [7] have been identified as urgent research priorities.

### Study Purpose and Objectives

The purpose of this study is to investigate the experiences of health system leaders and nurses during COVID-19 and to develop recommendations to inform pre-, during-, and postcrisis leadership strategies and practices for health systems leaders. The specific objectives are as follows:

1. Examine health system leadership challenges, enablers, and effective strategies at the organizational level in response to the evolving COVID-19 pandemic.
2. Examine nurses’ (registered nurse [RN] and registered psychiatric nurse or licensed practical nurse) experiences during COVID-19, their perceptions of leaders’ support for them and their working conditions, and the impact on their psychological health and well-being.
3. Investigate health system leaders’ promotion of their own health and how their leadership shaped nurses’ psychological health to mitigate strain and burnout.
4. Examine how health system leaders have fostered organizational resilience and how that may be shaped by gender.
5. Generate recommendations of leadership strategies to foster leader and staff psychological health and well-being and enhance organizational resilience.

An in-depth understanding of the factors facing leaders and staff throughout the pandemic and corresponding leadership strategies could provide several benefits. It could form the basis for individual, team, and organizational reflection, debriefs, and reviews of performance during the COVID-19 response. It also has the potential to inform emergency preparedness training, as well as guide crisis leadership practice during the ongoing pandemic and postpandemic context, as well as for future crises. Finally, the recommendations of strategies and practices based on this study’s findings could contribute to enhanced leadership and organizational performance and resilience more generally, partly by prioritizing leaders’ and nurses’ psychological health and well-being. Given the central role that health systems leaders played in the COVID-19 response and in advising government, with public health, and given the commonality of challenges that leaders in other sectors faced during this time, there is potential for this study’s recommendations to extend to other professional domains [38,39].

## Methods

### Study Design

To achieve this study’s objectives, we will use a qualitative exploratory inquiry [40] to explore the experiences of leaders and nurses. This design will enable us to answer the “what” and “how” associated with improving and advancing postcrisis leadership strategies and practices in hospital settings across 3 provinces. We will collect data from over 3 years across urban centers in 3 Canadian provinces using a multimethod approach consisting of individual semistructured interviews, focus group interviews (FGIs), data integration, and a nominal group technique to synthesize findings and develop recommendations.

### Conceptual Framework

**Figure 1:**
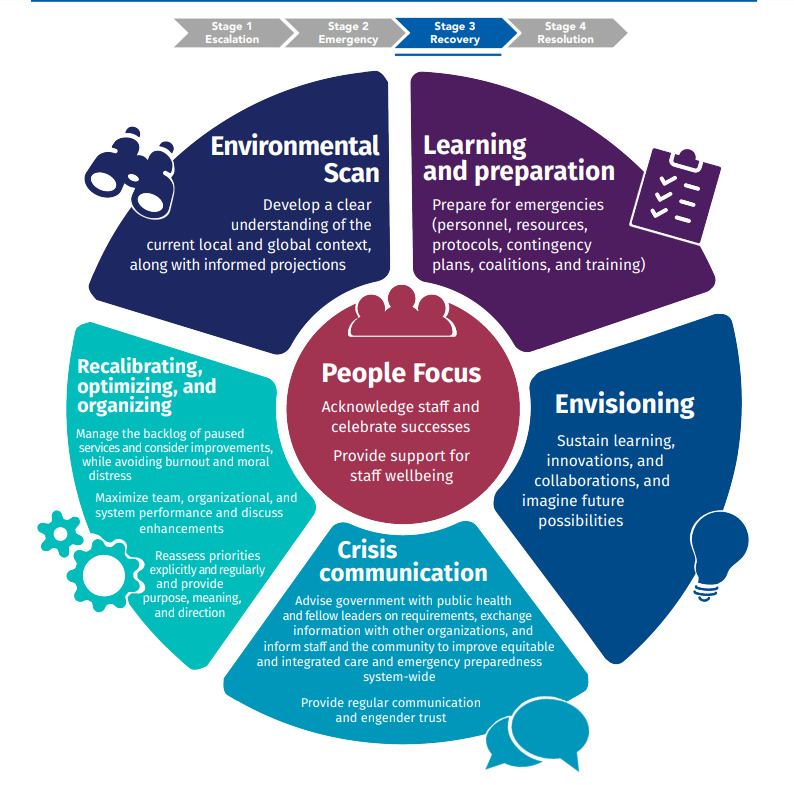
Geerts' framework of 10 leadership imperatives during the recovery stage of a crisis.

To ground our work, we draw from the evidence- and expertise-informed framework of imperatives for health system leaders conceptualized by a coinvestigator (JMG; Figure 1) [28]. This novel framework organizes the context of a crisis into four dynamic, progressive stages: (1) escalation, (2) emergency, (3) recovery, and (4) resolution [28], with defining characteristics and leadership imperatives presented for each stage. For example, leadership capabilities in the escalation stage involve rapidly gauging the nature, scale, and scope of an imminent threat, and preparing leaders and staff to implement preplanned organizational response protocols and gathering requisite resources [41,42]. The emergency stage is marked by focusing exclusively on urgent priorities, putting all nonessential operations on pause, maximum staff dedication, consistent adherence to emergency protocols using a command and control approach, balanced with frontline adaptive autonomy [42]. The recovery stage requires the greatest range of leadership capabilities because this stage involves balancing intense competing priorities, maintaining staff engagement, and minimizing burnout, within a prolonged and highly stressful, uncertain, volatile, and complex environment [28]. Finally, the resolution stage is marked by the leader’s focus on organizational learning and a willingness to share lessons learned with other organizations, while reconsidering how to build back and to address the toll the crisis has taken on people [41,42]. Our approach highlights how health system leaders can create conditions for a psychologically healthier workforce that can be sustained beyond the crisis.

The framework developed by Geerts et al [28] serves as the basis for gathering and organizing data about how health systems leaders implemented key leadership imperatives across the 4 stages during the COVID-19 pandemic, as well as how leaders sustained their own health and how their leadership shaped nurses’ psychological health and well-being and strengthened organizational resilience. The concepts of well-being and organizational resilience align with the leadership imperatives central to the framework, which guides leaders before, throughout, and following crises. Leaders who can implement these imperatives are ideally situated to anticipate and respond to urgent needs and collaborate with nurses to cocreate organizational conditions across all 4 stages that best serve nurses and the patients entrusted to their care.

### Management and Administrative Structure

This study will be led by SU and PEB, with support from study coauthors (also termed the “core team”). The team will also be supported by a study advisory committee (“AC”), composed of senior health care leaders, directors, middle managers, frontline managers, nurses, and stakeholder representatives from urban academic and community hospitals from each province (Alberta, Manitoba, and Ontario). They will be involved in assisting with the recruitment of participants, providing consultation, particularly about initial study recommendations, and facilitating knowledge dissemination. The administrative team will be made up of a research coordinator (RC), a data manager (DM), and a graduate research assistant.

### Settings

To increase the potential for variations in health care settings and transferability of findings, we purposefully selected 3 provinces with wide variations in COVID-19 incidence rates (Alberta 598,813, Manitoba 147,329, Ontario 1,163,243, as of August 13, 2022; Government of Canada) [43]. Additionally, the 3 provinces were among the 6 provinces in Canada with the lowest distribution of RNs per 100,000 population in 2021, whereby Ontario is at the bottom of the list with only 690, Alberta with 835, and Manitoba with 1037 RNs/100,000 population [44]. Similarly, the 3 provinces were among the top 5 provinces with the highest COVID-19–related mortality in 2021, with 10,206, 3263, and 1420 cumulative deaths for Ontario, Alberta, and Manitoba, respectively [45]. We will purposively select 1 academic and 1 community hospital from each of the 3 provinces to investigate how different provinces and leadership roles were enacted and how various factors (including unit overcapacity issues, leadership decision-making, administrative structures, and personal characteristics) may shape the work environment and the overall effects on the health and well-being of the leadership and nursing workforce. We will compare leader responses across provinces and compare leader responses with those of nurses from their same hospital to assess whether there is a disconnect in the frequency or effectiveness of leader practices.

### Participants and Sample Size

The sample will consist of 66-84 leaders (objectives 1 and 3) and 30-63 nurses (objective 2) as illustrated in Tables 1 and 2, and Table 3, respectively. When and how data saturation (ie, when new data adds only minor variations to categories) [46] can be achieved is difficult to determine before the onset of this study, as it depends on the volume and complexity of the data [40,47]. Based on similar qualitative studies with leaders, the proposed sample size should achieve saturation and will be robust enough to allow exploration within and across groups.

**Table 1:** Sample size of leaders to be interviewed per province by category and hospital type.

Hospital type	Category of leaders to be recruited			Total
	Senior leaders	Directors or middle managers	Frontline managers	
Academic	3-4	4-5	4-5	11-14
Community	3-4	4-5	4-5	11-14

**Table 2:** Total (Alberta, Manitoba, and Ontario) sample size of leaders to be interviewed by category and hospital type.

Hospital type	Category of leaders to be recruited				Total
	Senior leaders	Directors or middle managers	Frontline managers		
Academic	9-12	12-15	12-15	33-42	
Community	9-12	12-15	12-15	33-42	
Total	18-24	24-30	24-30	66-84	

**Table 3:** Sample size of nurses to be interviewed during focus group interviews by province.

Province	Nurses per FGI^a^ session	FGI sessions	Total nurses
Alberta	5-7	2-3	10-21
Manitoba	5-7	2-3	10-21
Ontario	5-7	2-3	10-21
Total	15-21	6-9	30-63

^a^FGI: focus group interview.

For the purposes of this study, we defined categories of leaders as follows: senior leaders are executive-level decision-making management personnel responsible for achieving organizational goals and strategic plans within the day-to-day operational management of the organization. Directors or middle managers are supervised by an organization’s senior leaders, supervise frontline managers, and have a role in practical improvements in health care delivery. These leaders can be registered nurses, allied health professionals, or other health-related disciplines. Frontline managers (who are registered nurses or other health-related disciplines) have patient, operational, and fiscal responsibility for their units and oversee direct care nurses and other care providers.

For objectives 1 and 3, through criterion sampling [42], the three levels of leaders who have an experiential knowledge of working in the COVID-19 crisis will be recruited. We will engage the AC to assist in maximum variation sampling with the aim of creating a demographically diverse sample across age, gender, and racial identity from each leader group per province.

### Recruitment

In consultation with the core team, this study’s principal investigators (PIs) will extend personal invitations to candidates to join the AC, ensuring that diversity, diversity of expertise, perspective, and geographical location are represented [48-51]. To recruit leaders and nurse participants (objectives 1, 2, and 3), we will use multiple recruitment strategies, including drawing on the core team networks and working closely with the AC in their respective provinces to invite participation of senior hospital administrators within the selected hospitals. We will provide representatives from potential sites with information about this study, including participant requirements, which can contribute to gaining hospital ethics approval and access approval. Once committed, each site representative will email prospective participants based on a template that we provide. Interested personnel will then contact this study’s PI or RC, who will respond with a consent form and participant demographic questionnaire. The consent form explains that participants agree to include their responses in published results in anonymized or aggregate forms, with no identifying information included that would compromise their anonymity. Once these forms are completed, the participant will be officially enrolled in this study, and we will schedule interviews.

To recruit knowledge users (objective 5), we aim to recruit 30 knowledge users in health care systems from the 3 provinces, and an additional 10 knowledge users from across Canada identified by the team and AC. Knowledge users are health leaders (senior, director, and frontline) from hospitals in urban, rural, or remote regions, nurses, policy makers, and health service researchers who will be able to make informed decisions about the research findings and apply them to programs or policies.

### Data Collection

A multimethod approach, informed by the conceptual framework of imperatives for health systems leaders, will guide the development of an integrated coding framework and data analysis. Individual interviews will be conducted using a semistructured interview guide (Multimedia Appendix 1) and will consist of questions centering on the organizational context (barriers, policies or guidelines, and resources), leadership strategies and practices (eg, communication or decision-making) that were effective (or not), and organizational resilience (leader or nurse well-being, and organizational learnings). Each interview will begin with exploring how the COVID-19 pandemic was perceived by the individual, followed by standardized questions and prompts, and will conclude with an open-ended question regarding final insights to generate further meaning and gain more clarity and understanding (objectives 1 and 3).

FGIs will be conducted with nurses from the included hospitals. There will be 2 to 3 FGIs in each province, as indicated in Table 3, each lasting approximately 90 minutes. A core team member will lead discussions in each group and concerning 3 areas informed by the conceptual framework: context (eg, their work conditions, their ability to provide safe, and quality care), perceptions of leadership effectiveness (eg, decentralized decision-making, communication, and level of engagement), and organizational resilience (eg, psychological health, ability to be innovative, and learnings). Concomitantly, the RC will document interactions among participants and observations of group dynamics, noting language, tone of voice, and nonverbal body language, including whether these appear to be consistent among genders. At the conclusion of each FGI, the lead researcher will summarize the main points and seek clarity on areas of ambiguity.

Data will be integrated from individual interviews with leaders and FGIs with nurses and will be used as a foundation for the 1-day forum. Following data integration, we will engage knowledge users from across Canada in a 1-day online forum (objective 5). The integrated data from individual interviews with leaders and FGIs with nurses will be used as a foundation for the forum. The nominal group technique is designed to obtain qualitative data from knowledge users to bring about consensus for promising postcrisis leadership strategies and practices [52,53]. This technique will be facilitated by engaging knowledge users in a 1-day online national forum via video technology (MS Teams [Microsoft Corp] or Zoom [Zoom Video Communications]) with the goal of achieving consensus for what should be shared and promoted nationwide. This technique has been proven effective in bridging the gap between researchers, policy makers, and practitioners [54-56]. Finally, an e-handbook will be developed for publication in collaboration with and mobilized by the AC and knowledge users.

### Ethical Considerations

This study is of low risk, and the participants are not considered vulnerable. This protocol has earlier been peer-reviewed prior to award (Multimedia Appendix 2); additionally, this protocol has been reviewed and approved by the University of Manitoba institutional review board, Office of Human Research Ethics (HE2023-0193), the University of Alberta institutional review board (Covenant Health Research Center, Pro-00136740), and the Hamilton Integrated Research Ethics Board at McMaster University in Ontario (Panel B 16790). Additionally, operational approvals have been granted by Shared Health (the provincial health regulatory body for the province of Manitoba) and Alberta Health Services, the provincial health authority. At the time of drafting this paper, we obtained operational approval from 2 hospitals in Ontario. All participants who agreed to participate in this study have signed written informed consents. The informed consent document has clearly explained this study’s procedure, the risk involved (minimum risk), and the right of the participant to withdraw from this study at any point. Participants’ privacy is fully protected by deidentifying participants, anonymization, and full delinking. Participants were compensated for their time with a CAD $20 (US $14.41) e-gift card (Walmart, Tim Hortons, or Amazon).

### Data Management, Analysis, and Quality and Security

The interviews and FGIs will be recorded, and verbatim transcripts will be generated. The RC and graduate research assistant will compare the transcripts against the recordings to ensure accuracy, and we will use a transcript-based analysis to increase rigor. We will allocate anonymous identifiers to transcripts, field notes, audit trail information, and demographic data. Data will be managed using MAXQDA (MAXQDA - Distribution by VERBI GmbH) qualitative software.

Thematic analysis will be used to identify and analyze themes (patterns of meaning) within the data to provide an interpretive explanation of health system leaders’ experiences during the evolving COVID-19 pandemic. This analysis will also enable us to understand how leaders promoted their own psychological health and well-being, and that of the nursing staff. We will apply Braun and Clarke’s [57] 6-phase approach to thematic analysis to enhance scientific rigor and ensure the research team is open to emerging codes and categories as analysis continues. Coding of responses will be deductive and organized according to the stages and imperatives within the conceptual framework; however, a complementary inductive approach will be used to capture important themes not represented in the framework. We will code line-by-line and supplement this with fieldnotes and analytic memos. After an initial independent review of a sample of the first transcripts by the coprincipal investigators and consultation with the creator of the framework (JMG), an initial coding framework will be developed. This initial coding framework will then be applied to an initial subsample of seven transcripts by the RC and the DM for two reasons: (1) to get a sense of the kinds of themes that are emerging in the data and to streamline the framework by removing redundant codes [58], and (2) to report preliminary results of the data to the rest of the core research team.

After the coding of the initial subsample is complete, the PIs, RC, and DM will meet and discuss any discrepancies or understanding of codes to achieve consensus, as well as suggestions for how to modify and optimize the coding framework. The final coding framework will then be applied to all transcripts. Constant comparison will be used to pay attention to the experiences of leaders across 3 levels and nurses from the same hospitals. The coding framework helps organize participants’ responses regarding effective leadership strategies and practices in the context of the 4 stages of a crisis, with a focus on promoting leaders’ and nurses’ psychological health and well-being and building organizational resilience. Contextual factors will be considered, including similarities or differences of leader experiences across provinces and across hospitals that include unit size, specialties (medicine or surgery, emergency department, and intensive care unit), academic or community hospitals, and available resources. We will create an audit trail to identify and justify early themes or codes, and decisions made to modify the framework to generate desired data.

We will share initial raw data findings, early themes, and corresponding qualitative responses with the research team and AC, including overarching similarities and differences, and data alignment (or not) with the conceptual framework, as well as any proposals or modifications that researchers have already made to the data collection instruments or coding framework. This discussion is to ensure that there is consensus in the analytical approach to bolster the robustness and quality of the results and study recommendations. Although consistency in data collection is important, equally so when researching a dynamic social phenomenon is adapting the methods based on initial responses when it appears that doing so will address the research objectives more effectively [47]. Consequently, the researchers will review field notes following each FGI and consider which emerging issues should be discussed and explored in subsequent FGIs, as the diversity of contexts (province and type of hospital), experiences, and perceptions of nurses attending the FGIs will likely enable unanticipated but important themes to emerge which are worthy of further exploration.

Objective 4 involves integrating data collected to address objectives 1, 2, and 3, in which each set (interviews, FGIs, and field notes) will be given equal weighting in the analysis [57]. Data will be combined and threaded into a visual summary of effective leadership strategies and emerging themes of how health systems leaders promoted their own health, and how their leadership shaped nurses’ psychological health and well-being in the workplace to mitigate strain and burnout, while building organizational resilience. To begin, we will review each dataset and identify “promising” lead codes for further analysis [59], which will then be reapplied in an iterative way to the data.

To uphold study rigor, we aim to establish trustworthiness through transparency and several other tactics [60]. Credibility includes inviting participants to review postinterview transcripts to ensure accuracy and completeness and incorporating any requested changes to review their transcripts, and providing comments on preliminary findings to strengthen the accuracy of interpretations, and through rich description and using illustrative quotes. Reflexivity will be logged by the PI, the RC, and DM to enhance their self-awareness of the phenomenon and improve the quality of this study. Transparency and dependability will be established by creating an audit trail documenting detailed records of the data collection process, details of coding, interpretive decisions, theme development, methodological decisions and modifications, meeting minutes, and data triangulation by interviewing a variety of participants.

Confirmability and objectivity will be met by independent coding and analysis of a portion of the data by the team (noted above) [61,62] and by several stages of incorporating an iterative feedback process until consensus is reached. Finally, given the ongoing pressures on health system leaders and nurses, we will strive to be flexible in accommodating participants’ contributions.

Data security and management will comply with institutional ethics and provincial privacy requirements.

## Results

As of September 6, 2024, this study has made significant progress in line with the approved timelines. Data collection has been completed for objectives 1 and 3 in Alberta and Manitoba and has commenced in Ontario. Table 4 shows the distribution of the interviewed from the 3 study provinces.

**Table 4:** Distribution of leaders interviewed.

Category of leaders		Academic	Community	Total
**Alberta**				
	Senior leaders	3	2	5
	Directors or middle managers	0	1	1
	Frontline managers	5	3	8
	Total	8	6	14
**Manitoba**				
	Senior leaders	0	1	1
	Directors or middle managers	3	2	5
	Frontline managers	4	2	6
	Total	7	5	12
**Ontario**				
	Senior leaders	0	0	0
	Directors or middle managers	0	3	3
	Frontline managers	2	0	2
	Total	2	3	5
**Total for the 3 provinces (Alberta, Manitoba, and Ontario)**				
	Senior leaders	3	3	6
	Directors or middle managers	3	6	9
	Frontline managers	11	5	16
	Total	17	14	31

FGIs began in November 2024, and we anticipate interviews will be completed by the fall of 2025 in all provinces. Data will be integrated from individual interviews with leaders and FGIs with nurses and will be used as a foundation for the 1-day forum (nominal group technique). We anticipate beginning data integration in the fall of 2025 and extending into early 2026. The nominal group technique will be conducted in the spring of 2026, and the final report will be written in the summer of 2026.

## Discussion

### Principal Findings

Although there is available literature on disaster preparedness [26] and leadership strategies in emergencies [27], to the best of our knowledge, there is a dearth of guidance for leaders during all 4 stages of a crisis, as described by Geerts [28]. Furthermore, few empirical studies exist on preparing health systems leaders following a major crisis, and what is available is often based on anecdotal lessons learned and proposed strategies for senior leaders [30,33,34]. The various waves of COVID-19 severely overwhelmed hospitals worldwide, many of which were notably overburdened before the pandemic [35,36], resulting in more than 7 million reported global casualties [63] and the most severe health human resources crisis of our generation [28]. The latter involved massive staff shortages and turnover, unrivalled rates of staff absenteeism, burnout, and psychological distress and trauma. Along with ramifications on nurses’ well-being, the destructive impact of these conditions depleted their engagement and ability to provide compassionate, quality care to patients, and compromised their capacity to address the surgical and diagnostic backlog. Health systems leaders have a “duty to care for their workforce” [9] and are tasked with prioritizing their people, while reimagining what leadership, staffing, and health provision should look like for systems transformation in the evolving global, national, and regional contexts.

A review of the literature identified several knowledge gaps. First, there is a limited understanding of how leadership strategies and practices apply and adapt across all 4 distinct stages of a crisis. Second, there is limited knowledge of how health system leaders sustain themselves and their staff, including nurses, through an extended crisis, and whether this is shaped by the gender of leaders and nurses. Third, there is limited understanding of how health system leaders can influence workplace contexts to sustain their own and their staff’s well-being and how they can ultimately bolster organizational resilience. Finally, there is a lack of understanding of how the crisis-resilience relationship provides direction in how health care organizations can prepare for, respond to, and rebuild stronger following the evolving and future health care crises. Similarly, although there is strong empirical support indicating that leadership development interventions can facilitate outcomes at individual and organizational levels [39,64], there is limited available evidence to guide the design, delivery, and evaluation of crisis-specific training programs. This information could also be applied to increase individual, team, and organizational adaptability, resilience, and innovation more generally.

Developing a deeper understanding of how leaders at different levels experience and perform effectively during various stages of a crisis is an underresearched priority area. Validating the evidence- and expertise-informed conceptual framework is an important foundation for addressing this research gap. This is the first study to apply the Geerts model [28] to guide primary data collection, analysis, and recommendations for all 4 stages. From this foundation, a qualitative exploratory inquiry [6] is a suitable research approach to elicit a detailed understanding of a range of experiences and behaviors [65], in this case, those of health care leaders and nurses related to effective leadership strategies to promote psychological health and well-being and build organizational resilience. This approach will allow us to explore new areas of inquiry while also providing a credible structure within which to organize findings and optimize strategies for health system leaders.

Our study aligns with developing and implementing a comprehensive, multifaceted approach to integrated knowledge translation and end-of-grant knowledge translation strategies [63]. Integrated knowledge translation has been the primary strategy for building relationships with our AC partners from the outset of the project to assist in the uptake of results to inform decision-making for hospital leaders, managers, and policy makers, and thereby, positively impacting nurses and patients [66]. We will aim for a broad impact of context-relevant approaches, coordinated planning, leveraging relationships, and technology to support dissemination and application of our findings to practice [67,68]. The AC has committed to providing input into preliminary and end-of-study findings for interpretation, relevance, and clarity, and to cocreating critical implementation strategies. Senior leaders will be consulted regarding dissemination strategies, including how to reach diverse audiences and different leader groups [69].

Finally, this study’s results will provide health systems leaders access to evidence-based strategies to strengthen and optimize their own psychological health and well-being and those of the nursing workforce, which can contribute to improved clinical outcomes. Additionally, the findings will directly inform and improve crisis preparedness, given anticipated future health crises, and prepare health systems leaders to be more effective across all 4 stages of a crisis, equipping them to ensure that patients receive high-quality care by nurses and other health care professionals in workplaces that prioritize their health and well-being.

### Conclusions

The results of this study will make an important contribution to the crisis leadership literature, as this is the first study aimed at providing evidence-informed effective leadership strategies framed within the Geerts model [28]. The findings will support practices that health system leaders can implement to foster their own and nurses’ psychological health and well-being and build organizational resilience. The benefits of this study will include evidence for effective health system leadership and support for nurses, crisis preparedness, and lessons from the pandemic to address leadership responses in the 4 stages of the crisis model. End-of-study dissemination strategies have been planned to broaden the impact of context-specific approaches to relevant stakeholders to support dissemination of findings to practice. Overall, this study will generate practical and potentially transferable strategies to contribute to how health system leaders can more effectively lead during a crisis that can improve nurse outcomes and the patients entrusted to their care.

## Appendix

Multimedia Appendix 1 Interview and focus group interview guides.

Multimedia Appendix 2 Peer-reviewer report from the Health Policy & Systems Management Research/Recherche sur la gestion des systèmes et la politique de la santé (Canadian Institutes of Health Research; CIHR/IRSC).

## Abbreviations

AC: advisory committee
DM: data manager
FGI: focus group interview
IRB: institutional review board
PI: principal investigator
RC: research coordinator
RN: registered nurse
