# Supplementary material for: Health System Leadership for Psychological Health and Organizational Resilience During the COVID-19 Pandemic: Protocol for a Multimethod Study
Source: JMIR Res Protoc. 2025 May 28;14:e66402. doi: 10.2196/66402 (PMC12159553; doi:10.2196/66402)
Supplement: Multimedia Appendix 1 [file resprot_v14i1e66402_app1.pdf]

<u>Semi-Structured Interview Guide for Health Leaders</u>

General statement: We are meeting today to talk about your experience as a health leader during COVID-19 and how you take care of your mental health and wellbeing as well as the wellbeing of nurses in your hospital so we can learn from this for ongoing and future responses to health care crises.

There are 2 parts for this interview:

Part 1: Examine health system leadership challenges, facilitators, and effective strategies at the organizational level in response to the evolving COVID-19 pandemic.

Part 2: Investigate health system leaders' ability to promote their own health and how their leadership shaped nurses' psychological health to mitigate strain and burnout.

Before we go into these parts I have some introductory questions for you:

Tell me about what happened as you prepared for the COVID-19 pandemic? That is before you got your first COVID-19 patient. (**Escalation phase**)

Tell me what happened when you were faced with the pandemic at your site? That is from the time you started getting COVID-19 patients to the peak of the situation. (**Emergency phase**)

Tell me what happened when the emergency phase of the pandemic was over but there was still a chance of a resurgence? **(Recovery**)

Tell me about the consequences of the pandemic, and how you experienced this? That is when the pandemic was weaning off and things started to return to normal (**Resolution**)

I will now start part 1 questions. Keeping in mind your responses to the previous questions, we will follow up more specific questions related to the pandemic.

Part 1: Health system leadership challenges and facilitators at the organizational level.

What were some of the barriers in the organization? What did you do to help your organization adapt to face the pandemic?
How did COVID-19 policies/guidelines shape how care was provided?
How was communication facilitated within your organization?
How did you make decisions to strike a balance between responding to the crisis and maintaining day-to-day operations?
What did you learn about how your organization managed the pandemic challenges?

I will now start part 2 questions.

Part 2: Investigate health system leaders' ability to promote their own health and how their leadership shaped nurses' psychological health to mitigate strain and burnout.

How did you begin preparing your staff for the COVID-19 response? How did you prepare yourself, if at all, as a leader in this pandemic?

Tell me how you supported nurses at the front lines of care?

In your leadership work, did you find yourself considering your staff's home responsibilities, and what consideration did you or were you able to give to these issues?

With respect to those leading you, did you feel supported in these areas? If so, how was this support provided?

Probe: Would you say the support was greater or more sympathetic or somehow different from women leaders than from men? (and could you give an example if so?)

How were you supported during the pandemic? How did you care for yourself during the pandemic?

Final question: Is there anything else you would like to share regarding your experiences and actions as a leader during COVID-19 that would be helpful for me to know?

Focus Group Interview Guide for Nurses

General statement: We are meeting today to learn more about how you cared for your own psychological health and wellbeing and what it was like to work during the pandemic. We want to learn about your experiences from this for ongoing and future responses to health care crises.

Objective 2: Examine nurses' (RN and LPN) experiences during COVID-19, their perceptions of leaders support for them and their working conditions and the impact on their psychological health and wellbeing.

Introductory question – begin discussion topic

Tell me what happened on your unit and/or the hospital as you prepared for the COVID-19 pandemic? That is before you get your first COVID-19 patient. (**Escalation phase**)

How did you react to the COVID-19 pandemic? How did other team members react to the COVID-19 pandemic?

Tell me about the changed that occurred on your unit/hospital when you were faced with the pandemic? That is from the time you started getting COVID-19 patients to the peak of the situation? (**Emergency phase**)

How did you get information you needed about the pandemic to provide patient care?
How did your manager adjust resources (Personal Protective Equipment (PPE), more staff) so you could continue providing care in a pandemic?
How did your manager remove 'red tape' so you could provide the patient care needed during the crisis? How did this influence your ability to care for your patients?
Were you encouraged to make decisions by taking advantage of your knowledge and skill to adapt and/or be creative as a result of the pandemic?

Tell me what happened when the emergency phase of the pandemic was over but there was still a chance of a resurgence? **(Recovery**)

Tell me about the consequences of the pandemic, and how you experienced this? That is when the pandemic was weaning off and things started to return to normal. (**Resolution**)

What are 1-2 main learnings from the pandemic you want your manager/leader to know that are needed for a better workplace and that would support your wellbeing so that you can provide safe, quality patient care?

What is the most meaningful strategy your leader did to support your health and wellbeing?
What would you have liked your manager/leader to do differently to better support you?
Did your leader consider any home responsibilities you had and what considerations were you given to these issues, if any? If so, can you provide an example.

Final question: Is there anything else I have not asked but would be helpful for me to know about supporting your mental health and wellbeing that I never asked but you would like me to know?
