# Supplementary material for: Health System Leadership for Psychological Health and Organizational Resilience During the COVID-19 Pandemic: Protocol for a Multimethod Study
Source: JMIR Res Protoc. 2025 May 28;14:e66402. doi: 10.2196/66402 (PMC12159553; doi:10.2196/66402)
Supplement: Multimedia Appendix 2 [file resprot_v14i1e66402_app2.pdf]

**Review Type / Type d'évaluation:**	Reviewer 1 / Évaluateur 1
**Name of Applicant / Nom du chercheur:**	Udod, Sonia
**Application No. / Numéro de demande:**	488150
**Agency / Agence:**	CIHR/IRSC
**Competition / Concours:**	Project Grant/Subvention Projet
**Committee / Comité:**	Health Policy & Systems Management Research/Recherche sur la gestion des systèmes et la politique de la santé
**Title / Titre:**	Advancing Health System Leadership Capacity for Psychological Health and Organizational Resilience during the COVID-19 Pandemic and Beyond

Adjudication Criteria/Critères de sélection

Initial Score/Cote Initiale: 4.4

Top/Bottom Selection/Groupe supérieur/inférieur

- (●) **Top/Groupe supérieur**
- ( ) **Bottom/Groupe inférieur**

**Review Type / Type d'évaluation:**	Reviewer 1 / Évaluateur 1
**Name of Applicant / Nom du chercheur:**	Udod, Sonia
**Application No. / Numéro de demande:**	488150
**Agency / Agence:**	CIHR/IRSC
**Competition / Concours:**	Project Grant/Subvention Projet
**Committee / Comité:**	Health Policy & Systems Management Research/Recherche sur la gestion des systèmes et la politique de la santé
**Title / Titre:**	Advancing Health System Leadership Capacity for Psychological Health and Organizational Resilience during the COVID-19 Pandemic and Beyond

Summary of Application/Résumé de la demande:

The application is a second resubmission.

The authors highlight that the COVID-19 pandemic has placed unprecedented demands on the healthcare system and its leaders. The authors stress that nurses are working in difficult conditions and are crushing workloads at the front line of care. They also state that nurses' health needs must be prioritized to mitigate a further workforce crisis. Authors also emphasize the fact that nurses were also particularly impacted because they are mainly women who often must balance home responsibilities with difficult working conditions. Moreover, the authors describe the importance of understanding healthcare leadership during a crisis and emphasize that there is currently a dearth of knowledge available on the recovery stage of crisis leadership. They specifically point to four important gaps:

1) Limited understanding of how leadership practices evolve and adapt across a trajectory from crisis to recovery in hospitals in Canada; 2) Limited knowledge of how health system leaders sustain themselves and nurses through an extended crisis, and how this is shaped by gender for leaders and nurses; 3) Limited understanding of how health system leaders can influence workplace contexts to improve their own and their staff's wellbeing that promotes organizational resilience; and 4) Lack of understanding of how the crisis-resilience relationship provides direction in how healthcare organizations adjust and respond to ongoing and future health care crises.

"The goal of this project is thus to understand the experiences of health leaders and nurses during COVID-19 and to develop recommendations to advance post-crisis leadership strategies and practices for Canadian health system leaders."

The focus of the grant is specifically on healthcare leaders and nurses' psychological health and wellbeing, as well as organizational resilience.

The researchers propose to use an exploratory inquiry to respond to five distinct aims with specific methods associated.

1) Examine health system leadership challenges, facilitators, and effective strategies at the organizational level in response to the evolving COVID-19 pandemic;
2) Examine nurses' (RN and LPN) experiences during COVID-19, their perceptions of leaders support for them and their working conditions and the impact on their psychological health and wellbeing;
3) Investigate health system leaders' ability to promote their own health and how their leadership shaped nurses' psychological health to mitigate strain and burnout;
4) Examine how health system leaders have fostered organizational resilience and how that may be shaped by gender;
5) Identify recommendations from the perspective of health system leaders and nurses that build psychological health and wellbeing and organizational resilience during the ongoing pandemic, post-crisis, and future crises to improve and advance health leadership and organizational performance.

To respond to each specific aim, the team will use specific methods:

Aim 1 and 3: The team will conduct 22-28 interviews with health leaders (senior, directors, frontline managers) per province.

Aim 2: The team will conduct 2-3 focus groups per province with 5-7 nurses in each to examine their work experiences during COVID-19

Aim 4: The team will integrate data from aim 1, 2 and 3

Aim 5: The team will use a nominal group technique to identify strategies, practices and recommendations that build organizational resilience and leader and nurses' well-being. They also plan a one day National Forum aimed at drafting promising post-crisis leadership strategies to then be disseminated largely across the country.

The study will take place in three provinces: Alberta, Manitoba, Ontario.

The results from this study will help advance leadership capacity of healthcare leaders and highlight best practices and strategies to promote organizational resilience, along with strengthening and optimizing their own and the psychological health and wellbeing of the nursing workforce. It will also help inform better preparedness for future crisis.

Strengths and Weaknesses/Forces et faiblesses:

Strengths:

The team incorporates 2 knowledge users from health authorities in Winnipeg, one site (Manitoba) where the study will be conducted. The team is composed of senior and mid-career investigators. The team has a previous history of working together on the topic of health leadership and have expertise with the proposed topic and the methods used in the proposal. Three researchers from the team are from each of the three selected provinces to facilitate recruitment and buy-in from hospitals.

According to the authors, no research has investigated the leaders and nurses' mental health and well-being at the recovery stage of a crisis which would fill an important gap in the literature and in the policy field. The authors present 4 important gaps that have been identified from the literature and how they intend to close these gaps with their grant.

The authors define and describe the important concepts presented in the proposal (i.e. organizational resilience, crisis leadership features, etc.) which is important to support the rationale of the study.

The team will use a specific framework (10 imperatives for health system leaders during the recovery stage of a crisis) that specifically is about health system leadership at the recovery stage of a crisis and describes how the framework will support the research project.

The project builds on previous qualitative findings about the emergency stage of the crisis for health leaders in Manitoba that was conducted by the applicants.

It is interesting that the team has elected to sample from large urban hospitals as well as community hospitals to see variations in leadership practices and nurses' well-being between these settings.

Criteria of rigor are well described by the authors in the grant (credibility, reflexivity, dependability, confirmability) and embedded in the methods.

The KT plan is strong and well-conceived. It will have a large reach across the country. An e-handbook will be written as a knowledge transfer strategy following the National Forum and the authors will have the e-handbook translated into French to reach francophone leaders and policymakers which is excellent. The team has already mobilized their Advisory committee in drafting the grant and this committee aims to support the dissemination of findings, which will likely increase its impact. The Advisory committee will have quarterly meetings and receive a monthly newsletter.

The letters of support support the application across the three targeted provinces.

The authors also responded appropriately to the reviewers' comments and incorporated many changes.

Weaknesses:
Although the letters of support cover the three provinces targeted in the project, no formal knowledge user is formally indicated as a KU in the grant for the province Ontario and for the province Alberta.

I wonder if that would have been useful to recruit nurses from the same department as the leaders that will be interviewed for Aims 1 and 3 of the study. This element is not completely clear in the grant but would having information from the leaders from a specific department and then having focus groups with nurses from the same department allow for a more comprehensive understanding of organizational resilience and crisis management. As such, some department could be perceived as a specific case, where some leadership strategies could be compared to nurses' experiences and well-being in the same department? I wonder if, a case study approach embedded in the same proposal could have been even more impactful outcomes.

Since the authors have included a gendered based analysis, it would have been interesting to have information about the current gender representation in leadership roles in healthcare. The authors clearly mention that nurses and frontline managers are mostly women, but it is unclear if this gender representation change when in higher leadership positions and how this could influence the analysis and results from the study.

The proposal was not always completely clear to read at times and would benefit for more conceptual clarity around "post-crisis" definition (question can be raised about if we really are in a post-crisis time).

**Review Type / Type d'évaluation:**	Reviewer 1 / Évaluateur 1
**Name of Applicant / Nom du chercheur:**	Udod, Sonia
**Application No. / Numéro de demande:**	488150
**Agency / Agence:**	CIHR/IRSC
**Competition / Concours:**	Project Grant/Subvention Projet
**Committee / Comité:**	Health Policy & Systems Management Research/Recherche sur la gestion des systèmes et la politique de la santé
**Title / Titre:**	Advancing Health System Leadership Capacity for Psychological Health and Organizational Resilience during the COVID-19 Pandemic and Beyond

Budget Recommendation/Recommandation budgétaire:

The budget seems appropriate.

Please indicate your appraisal of the integration of sex as a biological variable as a strength, weakness, or not applicable to the proposal./Prière de sélectionner une option pour donner votre évaluation de l'intégration du sexe comme variable biologique en tant que point fort ou point faible de la proposition, ou en tant qu'élément non applicable à la proposition.

- ○ **Strength/Point fort**
- ○ **Weakness/Point faible**
- ◉ **Not applicable/Non applicable**

Please indicate your appraisal of the integration of gender as a socio-cultural determinant of health as a strength, weakness, or not applicable to the proposal./Prière de sélectionner une option pour donner votre évaluation de l'intégration du genre comme déterminant socioculturel de la santé en tant que point fort ou point faible de la proposition, ou en tant qu'élément non applicable à la proposition.

- ◉ **Strength/Point fort**
- ○ **Weakness/Point faible**
- ○ **Not applicable/Non applicable**

Sex and/or Gender Considerations/Notions de sexe et/ou de genre:

The applicants have clearly described a diversity of ways in which gender roles and considerations should be taken into consideration in this project about health leadership in times of crisis.

The authors mention in the grant that they will use a sex and gender-based analysis to examine how gender impacts experiences and practices of men, women and non-binary individuals who are in position of leadership. In Aim 2, they mention that researchers will oversample the men in their sample because nurse are mainly women.

In publishing about the results, the team will provide specific input about gender differences and variations.

*** Note to the applicants to use the terms (woman, man) when discussing gender aspects and not (male, female) as these last terms are mentioned twice in the « Other project information box ».

**Review Type / Type d'évaluation:**	Reviewer 2 / Évaluateur 2
**Name of Applicant / Nom du chercheur:**	Udod, Sonia
**Application No. / Numéro de demande:**	488150
**Agency / Agence:**	CIHR/IRSC
**Competition / Concours:**	Project Grant/Subvention Projet
**Committee / Comité:**	Health Policy & Systems Management Research/Recherche sur la gestion des systèmes et la politique de la santé
**Title / Titre:**	Advancing Health System Leadership Capacity for Psychological Health and Organizational Resilience during the COVID-19 Pandemic and Beyond

Adjudication Criteria/Critères de sélection

Initial Score/Cote Initiale: 4.0

Top/Bottom Selection/Groupe supérieur/inférieur

- (●) **Top/Groupe supérieur**
- ( ) **Bottom/Groupe inférieur**

**Review Type / Type d'évaluation:**	Reviewer 2 / Évaluateur 2
**Name of Applicant / Nom du chercheur:**	Udod, Sonia
**Application No. / Numéro de demande:**	488150
**Agency / Agence:**	CIHR/IRSC
**Competition / Concours:**	Project Grant/Subvention Projet
**Committee / Comité:**	Health Policy & Systems Management Research/Recherche sur la gestion des systèmes et la politique de la santé
**Title / Titre:**	Advancing Health System Leadership Capacity for Psychological Health and Organizational Resilience during the COVID-19 Pandemic and Beyond

Summary of Application/Résumé de la demande:

The aim of this 3 year study is to understand the experiences of health system leaders
and nurses during COVID-19 and to develop recommendations that will advance post-crisis leadership strategies and practices for Canadian health leaders to address leader and nurses' psychological health and wellbeing, as well as organizational resilience.

Geerts' framework of 10 imperatives for health leaders during the recovery stage of a crisis will be used for a multi-methods study.

The study will be conducted in 6 hospitals in 3 provinces (2 per province) through the following methods:

1.Individual interviews with leaders

2.Focus groups with nurses

3.Interviews with leaders

4.Data integration

5.Nominal group technique to identify strategies, practices, to build psychological health and organizational resilience

Outcome: Synthesis of promising practices

**Review Type / Type d'évaluation:**	Reviewer 2 / Évaluateur 2
**Name of Applicant / Nom du chercheur:**	Udod, Sonia
**Application No. / Numéro de demande:**	488150
**Agency / Agence:**	CIHR/IRSC
**Competition / Concours:**	Project Grant/Subvention Projet
**Committee / Comité:**	Health Policy & Systems Management Research/Recherche sur la gestion des systèmes et la politique de la santé
**Title / Titre:**	Advancing Health System Leadership Capacity for Psychological Health and Organizational Resilience during the COVID-19 Pandemic and Beyond

Strengths and Weaknesses/Forces et faiblesses:

Strengths:

•The team is a strong one, with experience of working together over time.

•The focus on the recovery stage of the pandemic is positive

•The conceptual framework will be a useful one

•The researchers have well integrated comments from the last review.

•Studying nursing leadership in acute care hospitals in three provinces is a strength

•Progress shows legitimate delays.

•KT Plan is well thought through and stakeholders are appropriately involved

•Methods are appropriate and feasible

Areas for Improvement:

•The proposal could be improved in the Progress section, by saying more specifically how it builds on existing research and its findings. It is positive that there is no budgetary overlap. The Progress section makes the case for the researcher but would be stronger if it made the case for the specific focus of the research, especially given the phase of the pandemic.

•The focus of the proposal could be more precise and consistent throughout the proposal. For example, the Aim is stated in the summary as crisis leadership, while the aim in body of proposal is post-crisis leadership

• The argument for the research focuses on the challenges for nurses and the nursing workforce before and during COVID-19. The argument would be stronger with a greater focus on evidence of what comes into post-crises leadership and how it is different than in-crisis leadership.

•The proposal is not always easy to read, and the logical flow could be improved, with the removal of unnecessary repetition of points.

•Leaders' health and building organizational resilience post COVID (when new stresses and external-to the hospital forces buffet health care, specific organizations, and leaders) seem to be two different things.

•There seems to be an equation between individuals' "psychological wellbeing" and "influencing organizational resilience". It is important that the specific character of the connection is made more strongly throughout the proposal, otherwise they seem like two separate concepts and the focus of the proposal seems diffuse. The proposal would be stronger if they were either not put together, or addressed more clearly and consistently throughout.

•Given the definition of organizational resilience in the proposal, I would look for interview questions for the leader that get at how they: "identify potential problems in the environment, ensure resources, assume good decision making and actions, and minimize the negative impact of a disturbance". These actions seem to be translated into "ways to promote leader and staff psychological health and wellbeing". This disjuncture could be better explained.

•Good questions are proposed for the interviews and focus groups. However, the questions are primarily about being in the crises rather than descriptions of post-crisis or experiences of coming out of specific COVID crises.

**Review Type / Type d'évaluation:**	Reviewer 2 / Évaluateur 2
**Name of Applicant / Nom du chercheur:**	Udod, Sonia
**Application No. / Numéro de demande:**	488150
**Agency / Agence:**	CIHR/IRSC
**Competition / Concours:**	Project Grant/Subvention Projet
**Committee / Comité:**	Health Policy & Systems Management Research/Recherche sur la gestion des systèmes et la politique de la santé
**Title / Titre:**	Advancing Health System Leadership Capacity for Psychological Health and Organizational Resilience during the COVID-19 Pandemic and Beyond

Budget Recommendation/Recommandation budgétaire:

The budget is satisfactory

Please indicate your appraisal of the integration of sex as a biological variable as a strength, weakness, or not applicable to the proposal./Prière de sélectionner une option pour donner votre évaluation de l'intégration du sexe comme variable biologique en tant que point fort ou point faible de la proposition, ou en tant qu'élément non applicable à la proposition.

- ○ **Strength/Point fort**
- ○ **Weakness/Point faible**
- ◉ **Not applicable/Non applicable**

Please indicate your appraisal of the integration of gender as a socio-cultural determinant of health as a strength, weakness, or not applicable to the proposal./Prière de sélectionner une option pour donner votre évaluation de l'intégration du genre comme déterminant socioculturel de la santé en tant que point fort ou point faible de la proposition, ou en tant qu'élément non applicable à la proposition.

- ◉ **Strength/Point fort**
- ○ **Weakness/Point faible**
- ○ **Not applicable/Non applicable**

**Review Type / Type d'évaluation:**	Reviewer 2 / Évaluateur 2
**Name of Applicant / Nom du chercheur:**	Udod, Sonia
**Application No. / Numéro de demande:**	488150
**Agency / Agence:**	CIHR/IRSC
**Competition / Concours:**	Project Grant/Subvention Projet
**Committee / Comité:**	Health Policy & Systems Management Research/Recherche sur la gestion des systèmes et la politique de la santé
**Title / Titre:**	Advancing Health System Leadership Capacity for Psychological Health and Organizational Resilience during the COVID-19 Pandemic and Beyond

Sex and/or Gender Considerations/Notions de sexe et/ou de genre:

Gender is addressed quite well. There are a couple of non-substantiated statements that are confusing related to women are better in crises, etc. It would be better to know how these statements figure into the design.

**Review Type / Type d'évaluation:**	Reviewer 3 / Évaluateur 3
**Name of Applicant / Nom du chercheur:**	Udod, Sonia
**Application No. / Numéro de demande:**	488150
**Agency / Agence:**	CIHR/IRSC
**Competition / Concours:**	Project Grant/Subvention Projet
**Committee / Comité:**	Health Policy & Systems Management Research/Recherche sur la gestion des systèmes et la politique de la santé
**Title / Titre:**	Advancing Health System Leadership Capacity for Psychological Health and Organizational Resilience during the COVID-19 Pandemic and Beyond

Adjudication Criteria/Critères de sélection

Initial Score/Cote Initiale: 4.0

Top/Bottom Selection/Groupe supérieur/inférieur

- (●) **Top/Groupe supérieur**
- ( ) **Bottom/Groupe inférieur**

**Review Type / Type d'évaluation:**	Reviewer 3 / Évaluateur 3
**Name of Applicant / Nom du chercheur:**	Udod, Sonia
**Application No. / Numéro de demande:**	488150
**Agency / Agence:**	CIHR/IRSC
**Competition / Concours:**	Project Grant/Subvention Projet
**Committee / Comité:**	Health Policy & Systems Management Research/Recherche sur la gestion des systèmes et la politique de la santé
**Title / Titre:**	Advancing Health System Leadership Capacity for Psychological Health and Organizational Resilience during the COVID-19 Pandemic and Beyond

Summary of Application/Résumé de la demande:

The overarching goal of this research study is to understand the experiences of health system leaders and nurses during COVID-19 and to develop recommendations that will advance crisis leadership strategies and practices for Canadian health leaders to address leader and nurses' psychological health and wellbeing, as well as organizational resilience.

5 specific research objectives are specified. Using a framework elaborated through a Delphi panel outlining 10 imperatives for health system leaders during the recovery phase of the covid pandemic, 4 research questions (not listed as such but discussed in the conceptual framework section) will be addressed:

1.How did health leaders implement these 10 leadership imperatives?

2.How did leadership actions address leader health?

3.How did leadership shape nurses' psychological health and well-being?

4.How did leadership shape organizational resilience?

A qualitative "exploratory inquiry" study is proposed involving interviews with approximately 12 managers (including senior, middle and front-line) in each of 2 hospitals in each of 3 provinces (total: between 66 and 84 managers) as well as focus group interviews with nurses (2-3 groups of 5-7 participants in 3 provinces, total 9 focus groups). Finally, a one-day on-line national forum using a nominal group approach will be conducted.

**Review Type / Type d'évaluation:**	Reviewer 3 / Évaluateur 3
**Name of Applicant / Nom du chercheur:**	Udod, Sonia
**Application No. / Numéro de demande:**	488150
**Agency / Agence:**	CIHR/IRSC
**Competition / Concours:**	Project Grant/Subvention Projet
**Committee / Comité:**	Health Policy & Systems Management Research/Recherche sur la gestion des systèmes et la politique de la santé
**Title / Titre:**	Advancing Health System Leadership Capacity for Psychological Health and Organizational Resilience during the COVID-19 Pandemic and Beyond

Strengths and Weaknesses/Forces et faiblesses:

This proposal is a resubmission and applicants have provided responses to previous reviews. Previous reviews were very positive and responses are comprehensive.

Although I can generally appreciate and salute conceptual complexity in a research proposal, in this case, I find the proposal to be conceptually muddled. Applicants use concepts in what I find to be a cavalier manner. For example, objectives refer to the following concepts: effective leadership strategies, leader and nurses' psychological health, organizational resilience. Unfortunately, none of these concepts are operationalized. The draft semi-structured interview guide for Health leaders (appendix E) and the draft focus group interview guide for nurses (appendix F) are not likely to generate valid data to measure these concepts, or more generally to measure most of the concepts that are referred to in the research objectives and most particularly the leadership imperatives. I understand that in this exploratory inquiry approach, data collection is open-ended and the conceptual framework is used in the analysis phase to make sense of data. However, this requires rich and in-depth data collection and I am far from convinced that the proposed data collection instruments in the context of 45-60 minute individual interviews with managers will provide such rich data. Focus groups with nurses may provide somewhat richer data but they may still be insufficient.

Research objectives and interview guides are framed in the context of the emerging pandemic while the conceptual framework concern the recovery phase. Although I understand that "emerging" in the objectives may refer to "recovery" in the framework, interview guides refer to very early stages of the pandemic, probably more "emergency" phase than recovery phase.

**Review Type / Type d'évaluation:**	Reviewer 3 / Évaluateur 3
**Name of Applicant / Nom du chercheur:**	Udod, Sonia
**Application No. / Numéro de demande:**	488150
**Agency / Agence:**	CIHR/IRSC
**Competition / Concours:**	Project Grant/Subvention Projet
**Committee / Comité:**	Health Policy & Systems Management Research/Recherche sur la gestion des systèmes et la politique de la santé
**Title / Titre:**	Advancing Health System Leadership Capacity for Psychological Health and Organizational Resilience during the COVID-19 Pandemic and Beyond

Budget Recommendation/Recommandation budgétaire:

Budget is appropriate.

Please indicate your appraisal of the integration of sex as a biological variable as a strength, weakness, or not applicable to the proposal./Prière de sélectionner une option pour donner votre évaluation de l'intégration du sexe comme variable biologique en tant que point fort ou point faible de la proposition, ou en tant qu'élément non applicable à la proposition.

- ◉ **Strength/Point fort**
- ○ **Weakness/Point faible**
- ○ **Not applicable/Non applicable**

Please indicate your appraisal of the integration of gender as a socio-cultural determinant of health as a strength, weakness, or not applicable to the proposal./Prière de sélectionner une option pour donner votre évaluation de l'intégration du genre comme déterminant socioculturel de la santé en tant que point fort ou point faible de la proposition, ou en tant qu'élément non applicable à la proposition.

- ◉ **Strength/Point fort**
- ○ **Weakness/Point faible**
- ○ **Not applicable/Non applicable**

**Review Type / Type d'évaluation:**	Reviewer 3 / Évaluateur 3
**Name of Applicant / Nom du chercheur:**	Udod, Sonia
**Application No. / Numéro de demande:**	488150
**Agency / Agence:**	CIHR/IRSC
**Competition / Concours:**	Project Grant/Subvention Projet
**Committee / Comité:**	Health Policy & Systems Management Research/Recherche sur la gestion des systèmes et la politique de la santé
**Title / Titre:**	Advancing Health System Leadership Capacity for Psychological Health and Organizational Resilience during the COVID-19 Pandemic and Beyond

Sex and/or Gender Considerations/Notions de sexe et/ou de genre:

An appropriate discussion of these issues is include in the cover page of the application, although it is unclear to me how this would be really be operationalized.
